# Ajmalicine and Reserpine: Indole Alkaloids as Multi-Target Directed Ligands Towards Factors Implicated in Alzheimer’s Disease

**DOI:** 10.3390/molecules25071609

**Published:** 2020-04-01

**Authors:** Priya Kashyap, Vivekanandan Kalaiselvan, Robin Kumar, Suresh Kumar

**Affiliations:** 1 University School of Biotechnology, Guru Gobind Singh Indraprastha University, Dwarka, Sector 16C, New Delhi 110075, India; girlnamedpia@gmail.com; 2 Indian Pharmacopoeia Commission, Pharmacovigilance Laboratory, Sector 23, Raj Nagar, Ghaziabad, Uttar Pradesh-201002, India; kalaiselvan.ipc@gov.in (V.K.); robinkumar.ipc@gov.in (R.K.)

**Keywords:** Alzheimer’s disease, reserpine, ajmalicine, multi target directed ligand, Aβ, BACE-1, MAO-B, molecular docking, *rauwolfia serpentina*

## Abstract

Alzheimer’s disease (AD) is a multifactorial disorder characterized by exponential loss of memory and cognitive deficit involving several disease modifying targets (amyloid beta, beta-secretase, monoaminoxidase-B, and cholinesterase). The present study explores multi-target directed ligand approach using secondary metabolite reserpine (RES) and ajmalicine (AJM) obtained from *Rauwolfia serpentina* roots. Novel LCMS and HPLC methods were developed for identification and quantification of reserpine and ajmalicine. In vitro enzyme inhibition assays were performed to evaluate anti-cholinesterase, β-site amyloid cleaving enzyme (BACE-1) inhibition and monoamine oxidase-B (MAO-B) inhibition, further analyzed with in silico analysis. Anti-amyloidogenic potential was studied using anti-aggregation studies along with TEM and circular dichroism (CD) analysis. In vitro neuroprotective potential against Aβ toxicity and anti-oxidative stress was demonstrated using PC12 cell cultures. Reserpine is a more potent dual cholinesterase inhibitor than ajmalicine (IC_50_ values of 1.7 μM (AChE) and 2.8 μM (BuChE)). The anti-aggregation activity of reserpine (68%) was more than ajmalicine (56%). Both compounds demonstrated neuroprotective activity against Aβ42 (92%) and H_2_O_2_ (93%) induced toxicity in PC12 cells against controls. Phytocompounds also inhibited MAO-B and BACE-1 enzymes in concentration dependent manner. Molecular docking studies indicated the strong binding of compounds to the catalytic site of targets. This novel study demonstrated that reserpine and ajmalicine as a multi-target directed ligand that have disease modifying potential for amelioration of AD.

## 1. Introduction

Alzheimer’s disease (AD) is the most common neurodegenerative disease characterized by memory loss and cognitive decline. The increasing disease burden is estimated to double every 20 years according to the World Alzheimer Report 2016 [1]. The worldwide economic burden is approximately US $800 billion per year [2]. AD is a complex multifactorial disease involving several identified targets. According to amyloid hypothesis, β-site amyloid cleaving enzyme (BACE-1) cleaves amyloid precursor protein (APP) at its extracellular site followed by intra-membrane cleavage by γ-secretase releasing insoluble Aβ peptides (Aβ40 or Aβ42 residues) causing extracellular plaque formation in amyloidogenic pathway of AD. Aβ42 aggregation also induces oxidative stress in the central nervous system (CNS), which creates a feedback loop accelerating Aβ42 fibrillation further [3]. Another affected target in AD is cholinergic neurotransmission in CNS due to reduced choline neurotransmitter secretion [4]. Despite advanced AD researches, the current treatment strategies for AD mainly deal with providing symptomatic relief by improving cholinergic neurotransmission in CNS. Currently approved drugs for AD includes anti-cholinesterase, such as donepezil, galanthamine, and NMDA (*N*-methyl-D-aspartate) antagonists (memantine) [5]. At present, no disease modifying drugs are available for the treatment of AD. Due to multifactorial nature of AD, several therapeutic strategies were devised that include prevention and inhibition of Aβ42 aggregation and reducing oxidative stress in CNS [6]. In view of these, multiple targets were identified in AD such as Aβ42 peptide, acetylcholinesterase (AChE), butyrylcholinesterase (BuChE), BACE-1, monoamine oxidase-B (MAO-B), and reactive oxygen species (ROS). The current strategy involved a multi-target directed ligand (MTDL) approach where the drug molecule(s) can target key enzymes along with disease causing peptides involved in AD, simultaneously, with single or multiple compounds that might be useful in providing therapeutic relief to AD patients. The PC12 cells are used as in vitro model systems to induce toxicity by Aβ fibrils or H_2_O_2_ [7,8].

*Rauwolfia serpentina* has been mentioned in ancient Ayurvedic texts for its nootropic activity and treatment of various CNS disorders associated with psychosis, schizophrenia, insanity, insomnia, and epilepsy [9]. Previous studies reported several phyto-constituents present in *R. serpentina* extract such as yohimbine, ajmaline, reserpine, and serpentine [10]. The major secondary metabolites in the current study, are indole alkaloids; reserpine (RES) and ajmalicine (AJM). Reserpine previously reported having antihypertensive properties that can also cross blood brain barrier because of its lipid solubility nature, whereas ajmalicine has also been reported for its antihypertensive activity [11,12] The objective of the current study is to elucidate the multi-target drug ligand potential of reserpine and ajmalicine. The selected compounds demonstrated to bind with AD targets and imparts anti-cholinesterase, anti-amyloidogenic, antioxidant, and neuroprotective activity along with anti-BACE-1 and anti-MAO B potential.

## 2. Results

### 2.1. Metabolites Identification Using UHPLC-QTOF/MS

UHPLC-QTOF MS was used for metabolite analysis of *R. serpentina* hydro-alcoholic extract. Crude extract was analyzed for masses present, in both electron spray ionization (ESI) positive and ESI negative mode. The masses that were detected consistently as [M + H] and [M − H] ions were identified and matched with literature database and identity of metabolites was established (Table 1). ESI positive mode gave better metabolite profiling; as it detected maximum number of constituents therefore further analysis was carried out in ESI positive mode. There were few unmatched and thus unidentified masses, which consistently appeared in trace quantities in LCMS runs.

**Figure 1:**
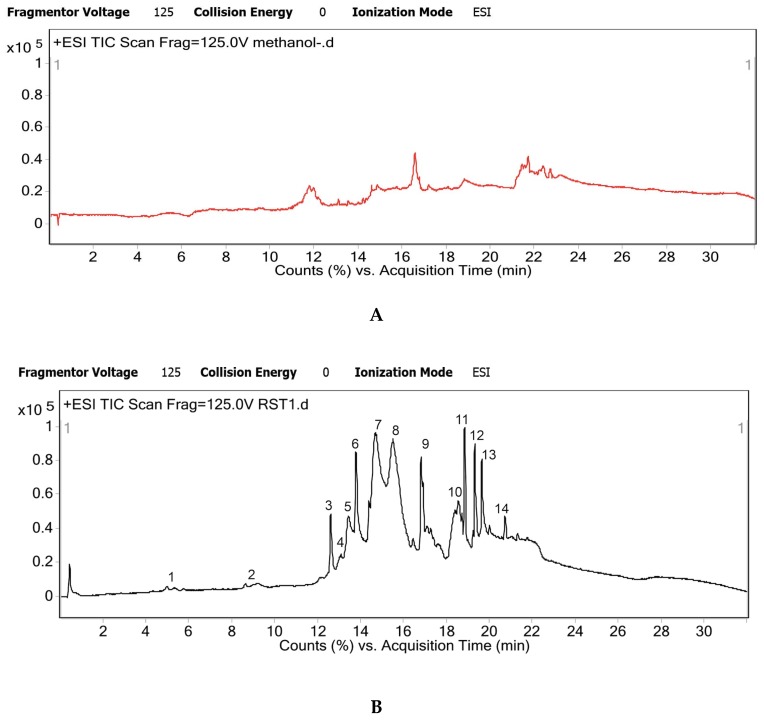
Total ion chromatogram generated by Agilent 6520 UHPLC-QTOF instrument and mass hunter software; (**A**) methanol blank chromatogram; (**B**) Hydroalcoholic root extract of *R. serepentina* chromatogram, *n* = 4 individual LCMS runs.

The individual peaks of chromatogram were resolved to the presence of a single metabolite, majorly proving the efficacy of the novel LCMS method developed (Figure 1). There were some trace impurity peaks present in the solvent (methanol blank) but it was ensured that these masses did not interfere with elution or profile of the desired metabolites (Figure 1A).

**Figure 2:**
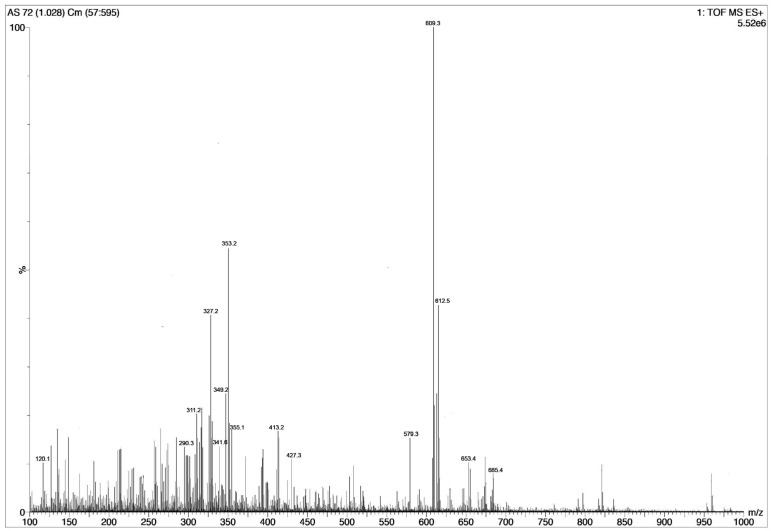
Total mass spectrogram of *R. serpentina* extract in positive mode ESI showing peaks of masses present. Highest *m*/*z* 609 reflects reserpine and *m*/*z* 353 reflects ajmalicine presence, *n* = 4 individual LCMS runs.

The total mass spectrogram (Figure 2) reported high counts of up to 5 × 10^6^ for RES and 2.5 × 10^6^ for AJM. Similarly, RES and AJM were found to be the major phytoconstituents present in *R. serpentina* extract with chromatographic peak area of 19% and 6%, respectively.

### 2.2. Reverse phase HPLC Method Development and Validation for RES and AJM Quantification

RES and AJM identified from LCMS were quantified using commercially purchased standards. A novel reproducible reverse phase HPLC (RP-HPLC) method for sensitive and rapid detection (or quantification) of RES and AJM was optimized for each, using economic solvents. The methods were validated according to “International Council for Harmonisation (ICH) of technical requirements for pharmaceuticals for human use” for stability, linearity, precision, limit of detection (LOD), and limit of quantification (LOQ) levels (Table 2) [13].

The calibration curve was plotted between absorbance and different concentrations (0.5–3.5 ppm) of test compound and linear regression equation was obtained. A correlation coefficient (R^2^) greater than 0.999 indicates good linearity thus the results showed an excellent correlation exists between the peak area and respective concentration of the compound (RES and AJM). Peak asymmetry greater than ‘1′ indicates peak tailing and less than ‘1′ indicates peak fronting. In the current study, both the methods developed have their peak asymmetry ratio around 1, which is a good measure for peak shape. Relative standard deviation (RSD) determination gives a validation for method precision. The RSD percentages for both the methods are low (RSD < 3%) therefore the RP-HPLC method precision is good. LOD and LOQ are calculated from signal-to-noise (S/N) ratio in multiples of 3 and 10, respectively. As a measure of sensitive method development, it was found that as minimum as 2.8 ppm for RES and 3.7 ppm for AJM could be successfully detected in the sample. Moreover, a least amount of 9.76 ppm for RES and 10.5 ppm for AJM could be successfully quantified in the sample using the developed method.

**Figure 3:**
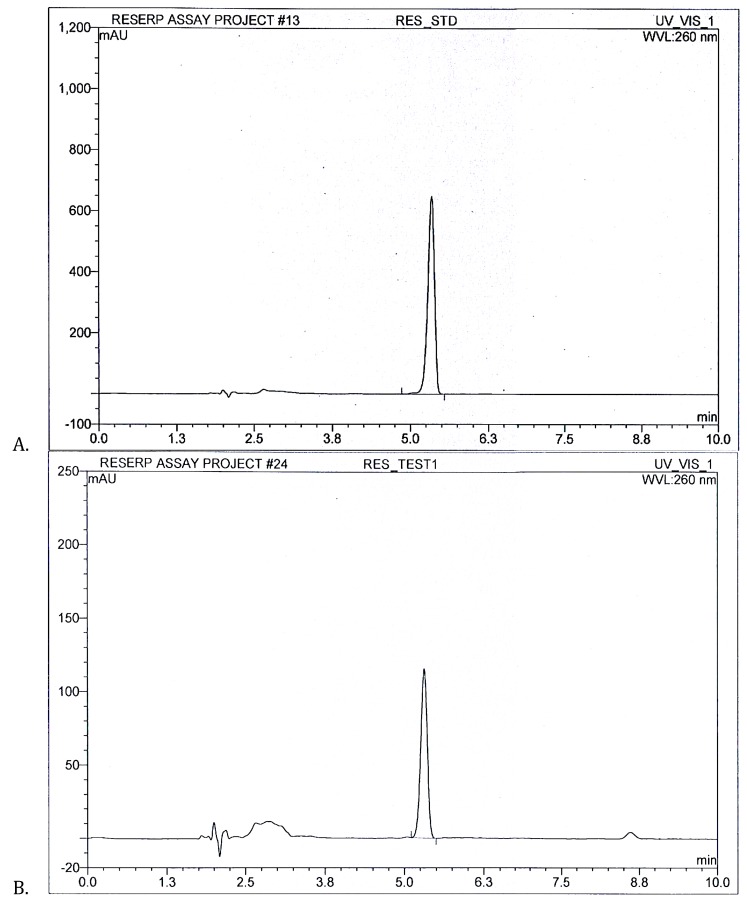
(**A**) RES standard peak (**B**) *R. serpentina* test extract resolved peaks at 5.3 min for RES quantification analysis using developed RP-HPLC method at 260 nm.

After successful RP-HPLC method development, respective compounds were quantified in *R. serpentina* extract. With six injections of standard, three individual injections of samples were chromatographed through the system. Figure 3 shows RES standard peak at 5.3 min with area 81.1 mAU/min alongside RES peak present in *R. serpentina* extract with area 13.9 mAU/min. RES amount was quantified as 15.42% *w*/*w* in *R. serpentina* extract.

**Figure 4:**
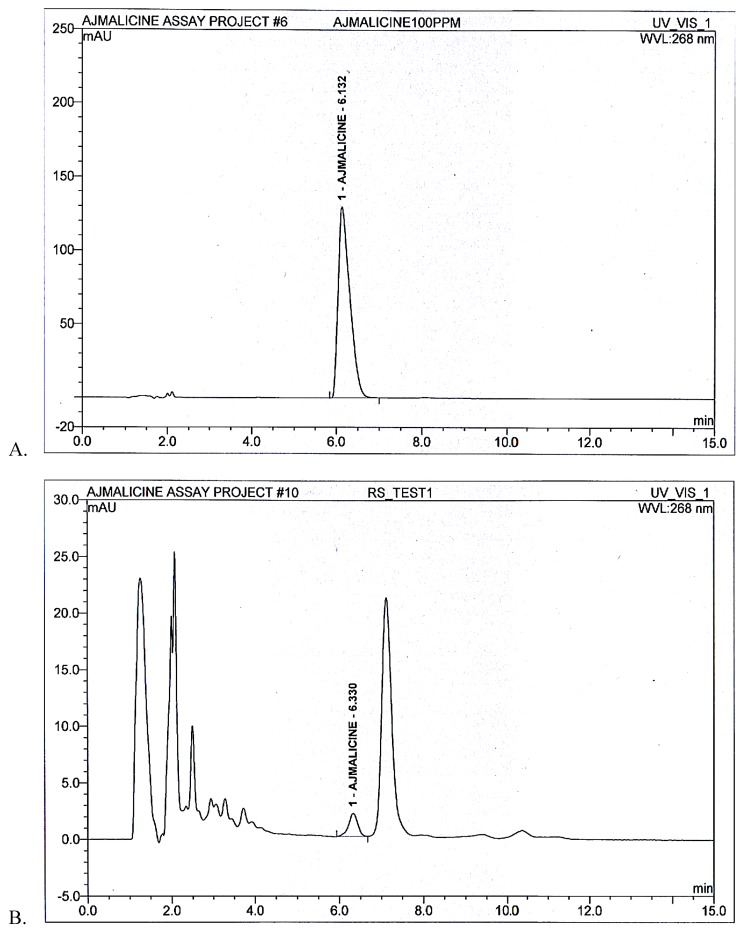
(**A**) Standard peak (**B**) *R. serpentina* test extract resolved peaks at 6.0 min and 6.3 min, respectively, for AJM quantification analysis using developed RP-HPLC method at 268 nm.

The area under curve for AJM standard peak at 6 min was determined to be 40.9 mAU/min while for AJM present in *R. serpentina* extract the area was calculated to be 0.52 mAU/min by Chromeleon 6.8 chromatography data system software (Figure 4). Thus, quantity of AJM was calculated to be 1.08% *w*/*w* in *R. serpentina* extract.

### 2.3. Anti-Amyloidogenic Effect of RES and AJM

**Figure 5:**
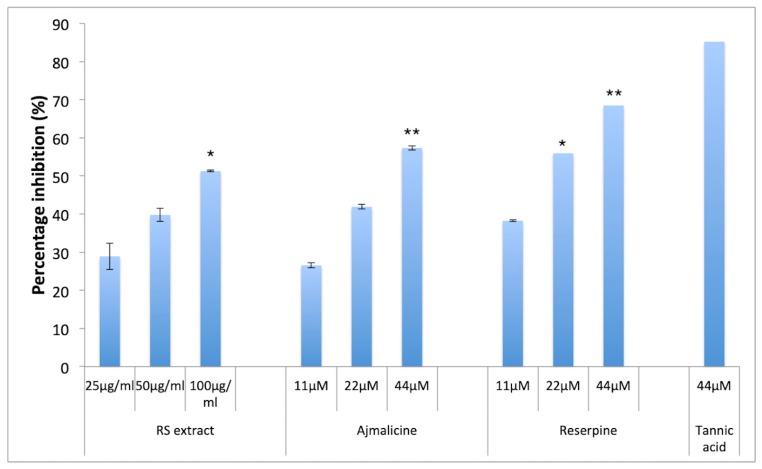
Percentage inhibition of Aβ42 aggregates formation; RS—*R.serpentina* extract (100, 50, 25 μg/mL); AJM—Ajmalicine (44, 22, 11 μM); RES—Reserpine (44, 22, 11 μM); NC—PC12 cells treated with Aβ42 only (40 μM); PC—tannic acid (44 μM), data represented as mean ± SEM, *n* = 3; and difference in mean is statistically significant (*) *p* < 0.05 and very significant (**) *p* < 0.01 as compared to control groups.

Amyloidogenesis involves aggregation of Aβ monomers into oligomers and proto-fibrils that leads to formation of plaques in CNS. The anti-aggregation potential of RES and AJM was analyzed through thioflavin T (ThT) fluorescence assay along with evaluation of red shift in Congo red (CR) dye binding assay. ThT probe gives bright fluorescence upon binding exclusively to Aβ42 proto-fibrils at 480 nm (emission) whereas free ThT quenches at the same excitation wavelength (450 nm), which makes it the most widely used probe against amyloid fibril detection. RES and AJM significantly inhibited the formation of Aβ42 fibrils in concentration dependent manner (11–44 μM), with 69% inhibition of fluorescence by RES (at 44 μM) (Figure 5) as compared to control sample (Aβ42 only). AJM and hydro-alcoholic root extract of *R. serpentina* demonstrated inhibition of aggregation (57% at 44 μM) and (51% at 100 μg/mL), respectively, as compared to control samples (Aβ42 only). An 85% inhibition of fluorescence was observed when Aβ42 incubated with tannic acid (positive control). The results indicate statistically significant anti-aggregation effect of RES and AJM.

**Figure 6:**
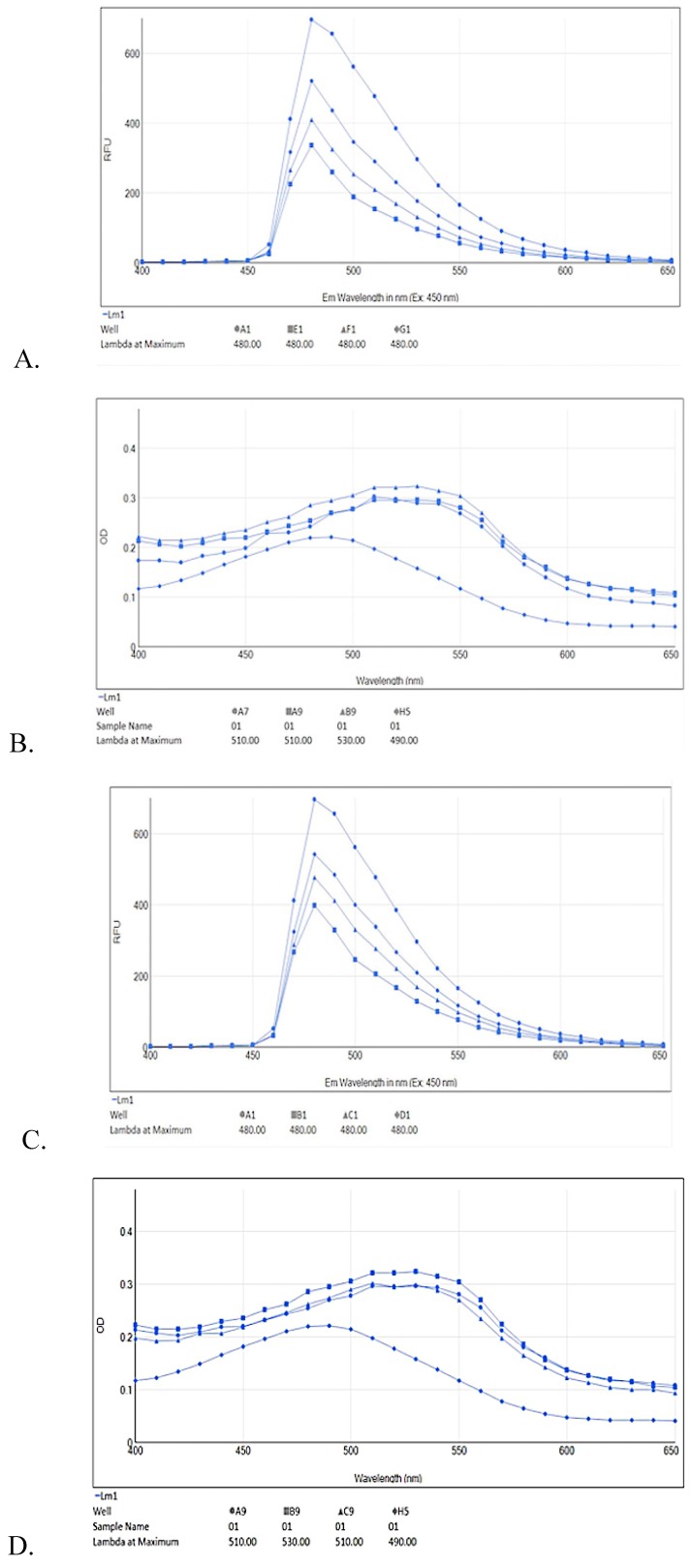

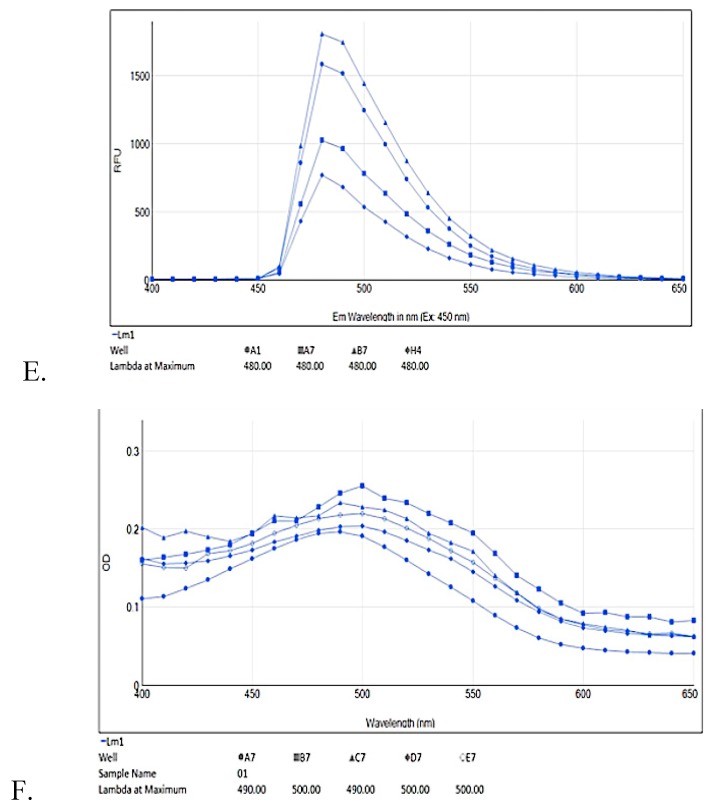
ThT fluorescence emission spectra (**A**,**C**,**E**) alongside red shift of Congo red dye (**B**,**D**,**F**) upon Aβ42 fibril formation with different test compound concentrations; RES **(6A)** ThT**:** A1—Aβ42 only, G1—Aβ42 + RES 11 μM, F1—Aβ42 + RES 22 μM, E1—Aβ42 + RES 44 μM; **(6B)** Congo red (CR): H5—CR only (490 nm), B9—Aβ42 only (530 nm), A9—Aβ42 + RES 22 μM (510 nm), A7—Aβ42 + RES 44 μM (510 nm). AJM **(6C)** ThT: A1—Aβ42 only, D1—Aβ42 + AJM 11 μM, C1—Aβ42 + AJM 22 μM, B1—Aβ42 + AJM 44 μM; **(6D)** CR: H5—CR only (490 nm) B9—Aβ42 only (530 nm), A9—Aβ42 + AJM 22 μM (510 nm), C9—Aβ42 + AJM 44 μM (510 nm). *R. serpentina* extract spectra **(6E)** ThT**:** B7—Aβ42 only, A1—Aβ42 + RS 25 μg/mL, A7—Aβ42 + RS 50 μg/mL, H4—Aβ42 + RS 100 μg/mL; and **(6F)** CR: A7—CR only (490 nm), B7—Aβ42 only (500 nm), C7—Aβ42 + RS 25 μg/mL (490 nm), E7—Aβ42 + RS 50 μg/mL (500 nm), D7—Aβ42 + RS 100 μg/mL (500 nm).

ThT fluorescence emission spectrum (400–650 nm) showed concentration dependent percentage inhibition of aggregation by test compounds (Figure 6A,C,E) supplement the ThT results mentioned above. Aβ42 fibrils showed fluorescence emission maxima at 480 nm. The test compound (10–40 μM) showed relative decrease in intensity of fluorescence emission spectra as compared to Aβ42 fibrils alone.

Congo red dye absorption spectra also support ThT determination of Aβ42 fibril inhibition in spectral shift assay. Upon Aβ42 interaction, CR undergoes a red shift in absorbance from 480nm to 500–550 nm. CR assay showed a typical absorbance at 490 nm whereas when Aβ42 added to CR the absorbance wavelength is shifted to 530 nm (Figure 6B,D,F). RES, AJM, and *R. serpentina* extract incubated with Aβ42 fibrils demonstrated reduced CR red shift close to 510 nm indicating strong anti-aggregation property of test compounds.

### 2.4. Evaluation of Inhibition of β Sheet Formation

**Figure 7:**
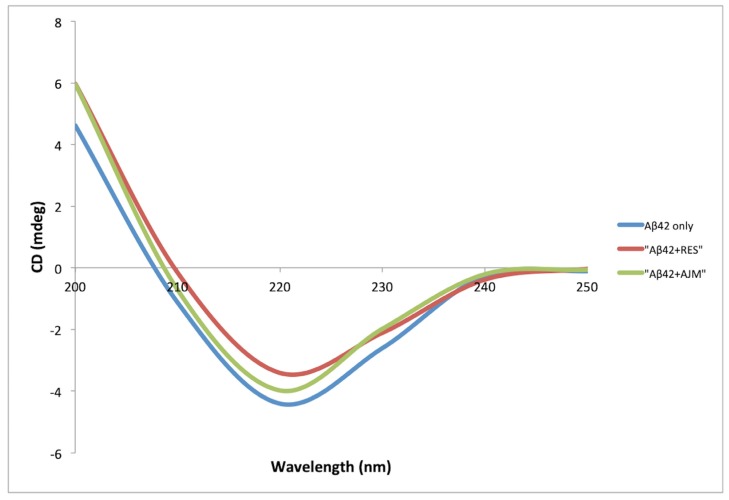
Circular dichroism (CD) spectra of Aβ42 (22 μM) alone (blue), Aβ42 with RES (22 μM) (red), and Aβ42 with AJM (22 μM) (green).

Aβ42 undergoes conformational changes during the process of oligomerization from random coil α-helix to structured β-sheets [14]. This change in the secondary structure is measured by circular dichroism (CD) spectroscopy in far UV region (200–250 nm), where the increase in negative bend near 215 nm is directly proportional to increased β-sheets content. CD spectra of Aβ42 co-incubated with RES and AJM showed that the process of Aβ42 oligomerization is strongly inhibited in the presence of the test compounds (Figure 7) as compared to control (Aβ42 only).

The secondary structure content (α-helix, β-sheets, and turns) was determined using CD spectra analysis software [14]. During Aβ fibrillation process, α-helix structure was found to be zero after 48 h incubation, while the β-sheet content increased to 75% in Aβ42 control (Table 3). RES demonstrated 64% inhibition in β-sheet formation whereas AJM inhibited the aggregation process to 53% as compared to control (Aβ42 only). This result is complementary to inhibition percentage of Aβ42 aggregation shown by ThT analysis.

### 2.5. Morphological Validation of Inhibition of Aβ42 Aggregates Formation

**Figure 8:**
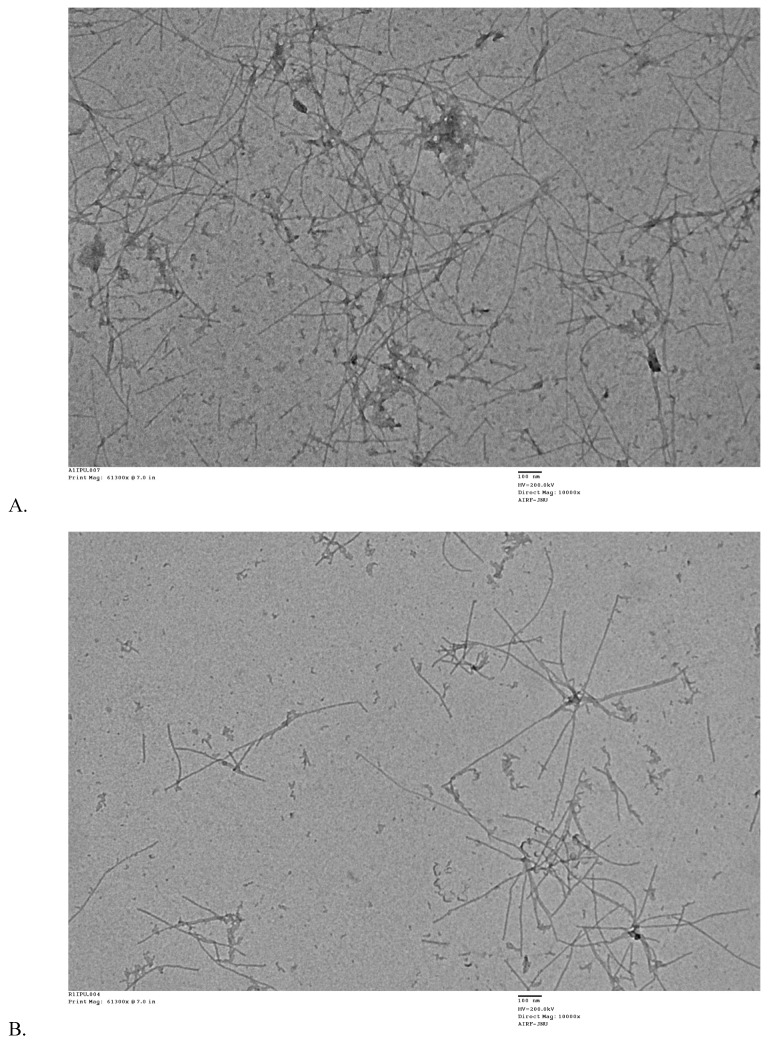

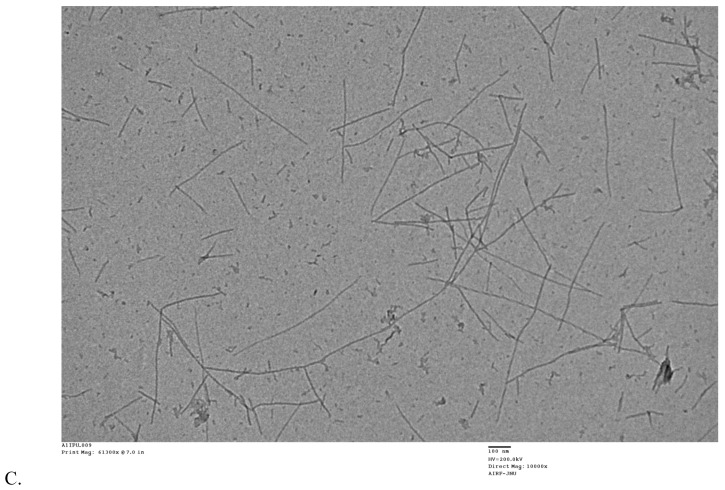
Transmission electron microscopy images of (**A**)—Aβ42 control incubated at 37 °C for 48 h; (**B**)—Aβ42 + 44 μM RES incubated at 37 °C for 48 h; and (**C**)—Aβ42 + 44 μM AJM incubated at 37 °C for 48 h. Images were presented at 100 nm.

The influence of RES and AJM on fibril formation was studied by analyzing the morphologies of Aβ42 aggregates by TEM. After 48 h of aggregation process, Aβ42 control showed abundant long, branched, and dense fibrils with peptide nucleation, which is characteristic of amyloids whereas RES and AJM shows shorter, loose, and less Aβ42 fibrils along with small oligomers (Figure 8).

### 2.6. RES and AJM Protects PC12 Cells Against Aβ42 Inflicted Cytotoxicity

Further investigation was done to study the cytotoxic effect of Aβ42 and protection against its neurotoxic effect by RES and AJM towards PC12 cells using MTT assay. PC12 cells resemble the phenotype of sympathetic ganglion neurons and are therefore used as model system to study neuroprotection against damages in AD [15]. Aβ42 is more toxic than Aβ40 oligomers in AD, therefore in the current study Aβ42 was used to induce toxicity. PC12 cells exposed to various concentrations of Aβ42 (2.5–80 μM) for 24 h showed concentration dependent cytotoxicity, evident by decrease in the rate of MTT reduction. Therefore, to study the protection by compounds, a fixed concentration of Aβ42 40 μM was used to induce damage towards PC12 cells.

**Figure 9:**
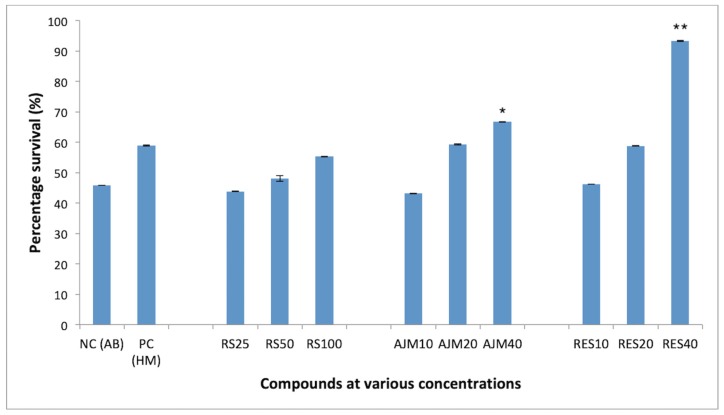
Concentration dependent percentage survival by MTT assay of Aβ42 fibrils (48 hrs) induced neurotoxicity on PC12 cells; RS—*R.serpentina* extract (100, 50, 25 μg/mL); AJM—Ajmalicine (40, 20, 10 μM); RES—Reserpine (40, 20, 10 μM); NC—PC12 cells treated with Aβ42 only (40 μM); PC—Homotaurine (40 μM), data represented as mean ± SEM, *n* = 3; and difference in mean is statistically significant (*) *p* < 0.05 and very significant (**) *p* < 0.01 as compared to control group.

PC12 cells showed significant concentration dependent survival in the presence of RES (92%) and AJM (67%) at equimolar concentration of Aβ42 (40 μM). *R. serpentina* extract also imparted 55% protection against Aβ42 cytotoxicity at 100 μg/mL which was comparable to the protection given by homotaurine (58%), used as positive control in the study (Figure 9).

### 2.7. In Vitro Neuroprotection Against Oxidative Stress

**Figure 10:**
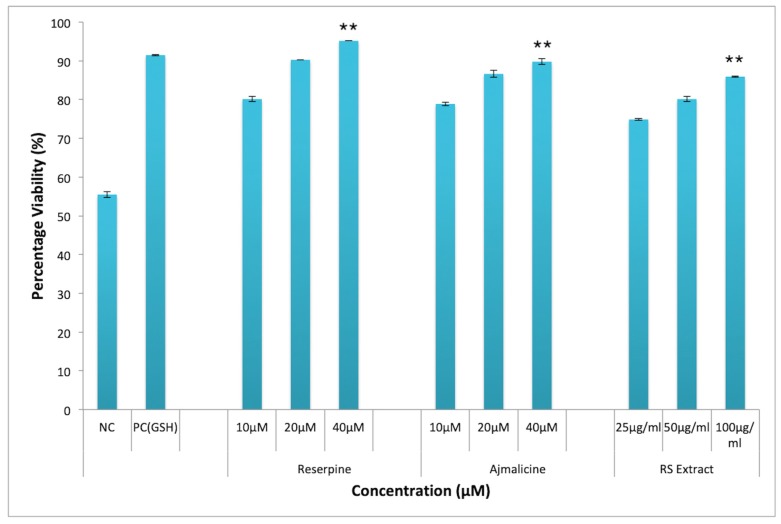
Neuroprotective effect against H_2_O_2_ induced cytotoxicity in PC12 cells; RES—Reserpine (40, 20, 10 μM); AJM—Ajmalicine (40, 20, 10 μM); RS—*R.serpentina* extract (100, 50, 25 μg/mL); PC: positive control (Glutathione, 40 µM); NC—negative control (H_2_O_2_ alone, 200 µM); values represents mean ± SEM, *n* = 3; and difference in mean is statistically significant (**) *p* < 0.05 as compared to control group.

Oxidative stress and generation of ROS have been shown to induce lipid peroxidation in cell membrane, which leads to cellular damage and subsequent cell death. Thus, the protective effect of RES and AJM against H_2_O_2_ induced oxidative stress towards PC12 cells was evaluated by MTT assay. Figure 10 showed significant protection against H_2_O_2_ cytotoxicity was observed in PC12 cells after pre-incubation with *R. serpentina* extract and its indole alkaloids RES and AJM. At extract concentration of 100 µg/mL highest viability was observed (86.4%) as compared to 31% viability in negative control group (H_2_O_2_ treatment alone). While PC12 cells had statistically significant results, surviving to 93% with RES (40 μM) treatment and to 89% with AJM (40 μM) treatment. The positive control glutathione (GSH) used in the study imparted 91% protection against H_2_O_2_ induced oxidative stress.

### 2.8. Evaluation of Dual Anti-Cholinesterase Potential of RES and AJM

**Figure 11:**
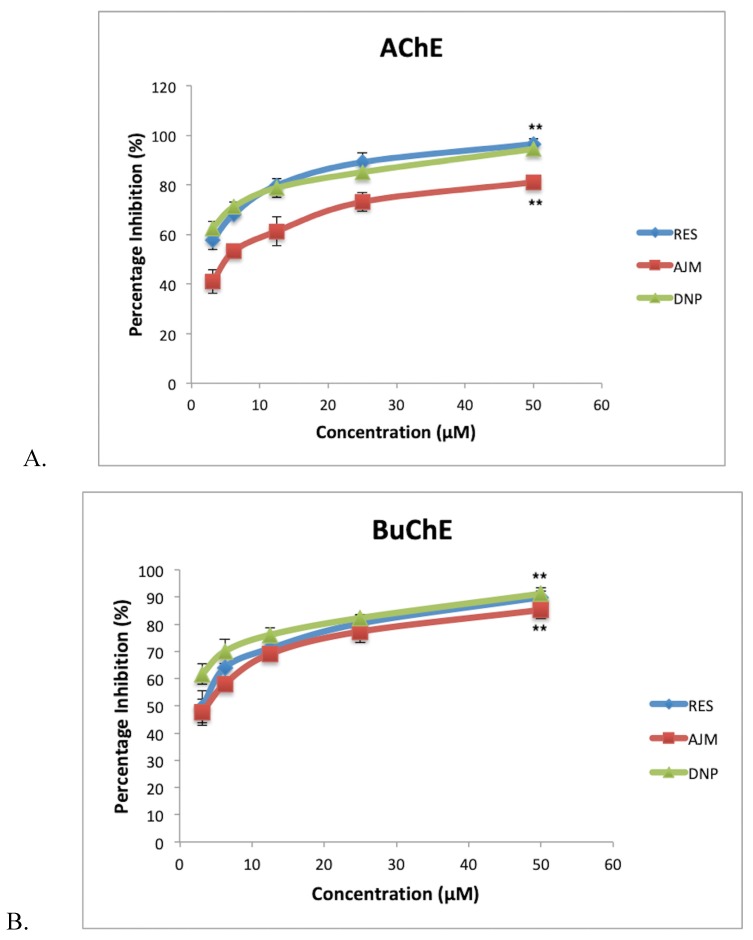
(**A**) acetylcholiesterase (AChE) percentage inhibition; (**B**) butyrylcholinesterase (BuChE) percentage inhibition at various tested concentrations of compounds (3.125–50 µM); RES-reserpine, AJM—ajmalicine, DNP—donepezil; values represent mean ± SEM, *n* = 3; and difference in mean is statistically very significant (**) *p* < 0.001 as compared to control groups.

Cholinergic neurotransmission is severely affected in AD, thus, to prolong neurotransmission a suitable drug compound must have anti-cholinesterase effect. RES and AJM achieved statistically significant dual anticholinesterase inhibition along with *R. serpentina* extract in concentration dependent manner (Figure 11). *R. serpentina* extract demonstrated 85% AChE inhibition at 200 µg/mL, post which a plateau response indicated enzyme saturation at 400 µg/mL (89% inhibition). RES at 50 µM concentration gave highest relative inhibition (96%), which is slightly higher than positive control donepezil inhibition percentage of AChE (94%). AJM showed 90% inhibition at 50 µM concentration. *R. serpentina* extract showed IC_50_ value of 14 µg/mL and 22 µg/mL for AChE and BuChE, respectively.

Table 4 indicates the IC_50_ values along with the selectivity of the inhibition by respective compounds for AChE and BuChE. Selectivity is the measure of inhibition for a particular enzyme over the other. In later stages of AD, BuChE levels increases in brain. BuChE catalyzes acetylcholine as it’s substrate. Therefore, the BuChE inhibition becomes important along with AChE inhibition. Although RES and AJM have selectivity for AChE, the results suggest almost equivalent inhibition for BuChE.

### 2.9. β-Site Amyloid Precursor Protein Cleaving Enzyme 1 Inhibition

**Figure 12:**
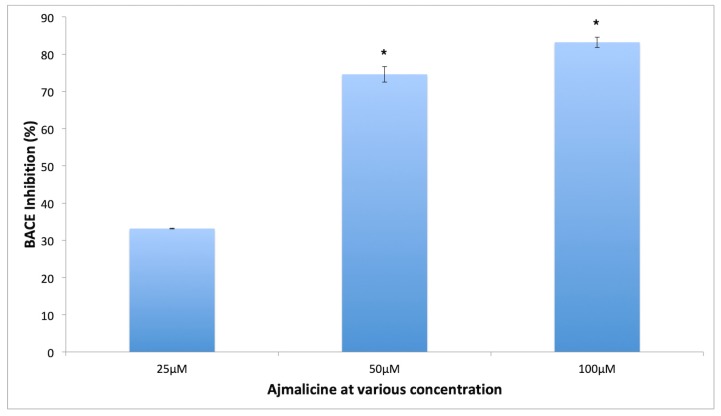
Concentration dependent β-site amyloid cleaving enzyme (BACE-1) inhibitory activity of AJM; values represent mean ± SEM, *n* = 3; and * difference in mean is statistically very significant *p* < 0.001 as compared to control group.

The BACE-1 enzyme inhibition has been shown to reduce the amount of pathogenic Aβ42 formation, which makes it one of the important targets for AD [16]. The results of the current study demonstrate the efficacy of lead compounds to inhibit the enzyme effectively (Figure 12). AJM showed the maximum inhibition of BACE-1 activity to 69% at 50 μM concentration whereas RES imparted 47% inhibition at same concentration. Therefore, AJM inhibitory potential was evaluated at various concentrations and AJM showed statistically significant BACE-1 enzyme inhibition in concentration dependent manner at various concentrations tested (25, 50, 100 μM) (Figure 12).

### 2.10. Inhibition of Monoaminoxidase-B Enzyme

**Figure 13:**
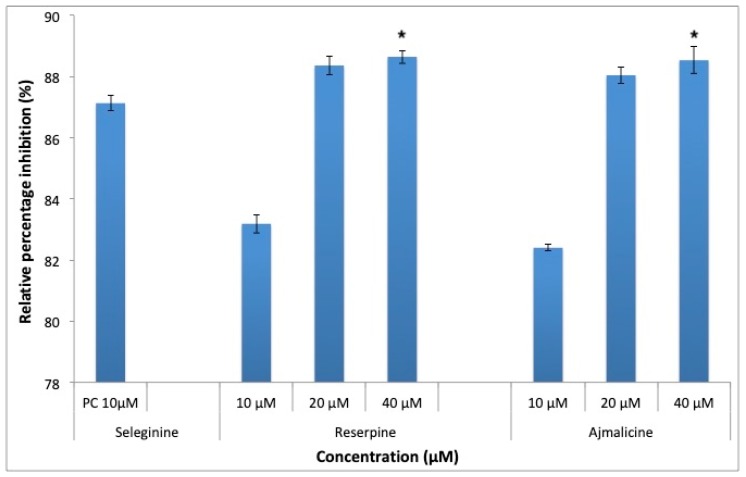
Monoamine oxidase-B (MAO-B) inhibition by positive control (selegiline) at 10 μM, RES and AJM at 10, 20 and 40 μM concentration; values represents mean ± SEM, *n* = 3; and * difference in mean is statistically very significant *p* < 0.001 as compared to control group.

MAO-B is responsible for production of toxic oxygenated free radicals, which is responsible for neurodegeneration in AD. RES and AJM significantly inhibited the MAO-B enzyme in a concentration dependent manner, at 10 μM, both the tested compounds gave comparable inhibition for MAO-B enzyme, 89% (40 μM) inhibition (Figure 13) was shown at higher concentrations compared to control.

### 2.11. Molecular Interaction of RES and AJM with AD Targets

Molecular docking revealed interaction of RES and AJM with catalytic site residues of studied AD targets (Aβ42, AChE, BuChE, BACE-1, and MAO-B) giving insight of the mechanism of inhibition of respective enzymes and peptide that correlate with in vitro studies. Molecular docking analysis (Table 5) clearly indicates that RES and AJM are excellent multi-target directed ligands capable of inhibiting Aβ42 aggregation and key enzymes implicated in AD such as AChE, BuChE, BACE-1, and MAO-B. RES and AJM strongly interacted with Aβ42 key residues with very low binding energy. Lower binding energy implies stable protein-ligand complex, thus, strong interaction. Based on the binding energies RES and AJM demonstrated better Aβ42 anti-aggregation potential as compared to positive control (tannic acid). Similarly, RES and AJM are better AChE and BuChE inhibitors as compared to positive control (galanthamine and tacrine) attributed to low binding energies signifying strong and stable interaction. AJM interacted with MAO-B catalytic site residue through hydrogen bond while RES interacted with comparatively weak hydrophobic environment suggesting AJM is better MAO-B inhibitor than RES.

### 2.12. Pharmacokinetics Analysis

Pharmacokinetics analysis for ADMET, of identified phytoconstituents showed that AJM proves to be a more ideal drug molecule as it obeys Lipinski rule of five along with suitable profile to cross the blood brain barrier (BBB) (Table 6). While RES failed to obey Lipinski rule of five majorly because of its molecular weight (above 500 g/mol) but showed good potential to cross the BBB. All the positive controls showed ideal drug profiles.

## 3. Discussion

Pathophysiology of AD is attributed to diverse, complex factors such as cognitive decline, memory loss, and extracellular plaque formation along with neurofibrillary tangles. From the inception of amyloid cascade hypothesis to its reappraisal, it is considered to be the primary cause of this form of senile dementia [17,18]. With the advent of research and new disease progression insights, now we can assign more targets with responsibility towards either pathophysiology or associated symptoms [5]. Despite major pharma-research focused on discovering novel disease-modifying molecules, the current treatment for AD targets its affiliated symptoms only [6]. In accordance with the aforesaid fact, we investigated the potential of natural compounds from *R. serpentina* (reserpine and ajmalicine) for their MTDL properties.

Naturally occurring alkaloids have long been used in therapeutic strategies against AD, such as galanthamine and huperzineA that are used in providing symptomatic relief to AD patients [19]. *R. serpentina* is abundant in many indole alkaloids such as reserpine, serpentine, rauwolfine, sarpagine, ajmaline, yohimbine, and ajmalicine [10]. In the current study the most abundant phytoconstituents identified in our test extract by UPLC-QTOF method were RES and AJM. Previous studies have shown similar identification of secondary metabolite profile of *R. serpentina* by UPLC-UV [10,20]. RES and AJM were quantified by a novel method developed and validated for RP-HPLC using an UV detector. This method is rapid and precise as they give reliable elution at early retention time using organic mobile phase as compared to previously reported methods [21,22]. The RP-HPLC method used in the current study has many advantages as compared to previously reported methods. These advantages are low LOD and LOQ as it offers sensitive detection and quantification of RES and AJM in trace amount. 

Aβ42 is a 4KDa fragment of mature APP, which self-aggregate to form toxic Aβ42 oligomers and finally proto-fibrils that are considered as a main pathological factor implicated in AD [23]. Compounds, which could inhibit Aβ42 aggregation or reverse this process of peptide nucleation would be of great therapeutic interest. Current study results showed concentration dependent, statistically significant anti-amyloidogenic potential of RES, AJM, and hydro-alcoholic root extract of *R. serpentina*. The exact mechanism of Aβ42 oligomerization to insoluble plaque formation is not clear but the secondary structure conformation studies suggest that β-sheet content is increased in Aβ42 oligomerization [24]. CD analysis results were in accordance to this observation as the reduced percentage of β-sheet content in RES and AJM treated samples of Aβ42 showed equivalent inhibition percentage in Aβ42 self-aggregation. The fact is further validated with electron micrographs showing short, unbranched fibrils and no peptide nucleation in RES and AJM treated samples of Aβ42 as compared to control (Aβ42 only). Insight of the inhibition mechanism could be understood by observing interactions at the molecular level. As evident from molecular docking analysis, RES, and AJM stacks themselves in between adjacent β-sheets and therefore inhibit further oligomerization. RES forms hydrogen bond with key residue (Asp23) and interacted hydrophobically to Lys 28 of Aβ42, bound internally in the steric zipper composed of KLVFFA (residues 16-21). Besides, RES also oriented itself to cover the hydrophobic core residues (Leu17, Val18, Phe19, and Phe20) of Aβ42, stabilizing the complex further. Aβ42 assembly requires Asp23 of one monomer to form a hydrogen bond with Lys28 of another monomer therefore to conclude RES can potentially inhibit Aβ42 aggregation. Furthermore, RES gave a binding score comparable to positive control compound (tannic acid). AJM interacts more with hydrophobic core inside steric zipper via hydrophobic interactions. It also bound to Asp23 with one hydrogen bond therefore it covers the residue (Asp23) required for oligomerization and hence inhibited Aβ42 aggregation process. Aβ42 oligomerization has been shown to accelerate upon AChE binding to Aβ42 by hydrophobic environment near peripheral active site (PAS) of AChE [25]. The indole ring of RES formed hydrogen bond with catalytic anionic site (CAS) residues Glu334 along with another hydrogen bond with Tyr72 in PAS of AChE. Similarly, AJM interacted with hydrogen bond towards Tyr286 (PAS residue) and Phe295-Phe297 in acyl binding pocket of CAS of AChE. Therefore, RES and AJM successfully occupy both the CAS and PAS of AChE, thereby inhibiting the enzyme as well inhibit AChE induced Aβ42 aggregation. The molecular docking results are well complemented with in vitro results of enzyme inhibition assay. 

In advanced cases of AD, BuChE secretion increases responsible for hydrolysis of AChE as it’s substrate. This accelerates the cholinergic decline process further. RES and AJM counteract the decrease in neurotransmitter levels (AChE) by significantly inhibiting BuChE in concentration dependent manner evident from in vitro enzyme inhibition assay. The molecular interactions showed strong binding energies of RES and AJM with BuChE, suggesting stable protein–ligand complex. Tacrine (known inhibitor) had lower docking score then RES and AJM. RES forms strong hydrogen bond with BuChE CAS residue (Val288) and occupies the PAS by interacting with a residue (Asn68) which lies near PAS (Asp70). Whereas AJM binds directly to CAS and PAS residues (Trp82 and Asp70, respectively). Thus, RES and AJM could prove to be better anti-cholinesterase drug ligands for therapeutics towards AD.

BACE-1 enzyme structure is found to exist in three different conformations based on a flap like secondary structure, which covers the active site of the enzyme. The BACE-1 structure (PDB ID 4D8C) used in the current study is closed flap structure, which is the dominant form exists upon binding to its substrate. Molecular docking analysis showed that the indole ring of RES interacts with the main catalytic site residues of the BACE-1 enzyme. The indole ring of RES acts as a hydrogen bond donor for Asp32, forming double hydrogen bond with catalytic site residue of BACE-1 while the isoprene tail of RES forms hydrophobic interaction in the catalytic gorge of BACE-1. On the other hand, AJM binds more strongly to BACE-1 with all hydrophobic interactions as it gave a high binding score with BACE-1. Probable explanation could be the stability AJM provides to the hydrophobic catalytic gorge of BACE-1 due to its compact size and orientation then RES. These molecular interactions are complemented with in vitro results showing statistically significant inhibition of BACE-1 enzyme, where observed potency of AJM is more than RES.

MAO-B enzyme plays an important role in generation of free radicals thus imparting oxidative stress at cellular level. RES and AJM inhibited MAO-B enzyme in concentration dependent manner comparable to positive control (selegiline). The in vitro results are complimented by molecular docking analysis where RES was observed to interact with the key catalytic site residues of MAO-B while AJM occupies the catalytic site by interacting with other residues there. RES interacted with Tyr326 and Ile199 by hydrogen bonding. AJM gave high binding score then RES probably because AJM formed more strong hydrogen bonds with residues surrounding catalytic site covering the main residues involved in hydrolysis of the substrate. The ADMET profile of AJM showed promising results of being able to act as ideal drug molecule with BBB permeability while RES structure could be utilized to develop active MTDL drug candidate with small molecular size.

## 4. Materials and Methods

### 4.1. Materials

LC-MS grade water, methanol, acetonitrile, formic acid, Zorbex UPLC C_18_ Silica Column, Agilent 6520 QTOF instrument, acetylcholinesterase from electric eel, butyrylcholinesterase from equine serum, amyloid beta protein (Link biotech), thioflavin T, Congo red dye, reserpine, and ajmalicine were purchased from Sigma Aldrich, India. MAO-B inhibition kit by Biovision. BACE-1 activity assay kit by Sigma Aldrich. RPMI 1640 medium, FBS, PBS, and other tissue culture grade chemicals were purchased from Himedia, India. PC12 (rat pheochromocytoma) cell line was obtained from NCCS, Pune, India. All other reagents used were of analytical grade. All preparations used in tissue culture experiments were filtered through 0.45 µm Axiva 25 mm CA filter. Ethical approval was obtained from GGS Indrapratha University for all the cell culture work done in proper confined facilities.

### 4.2. Preparation of Hydro-Alcoholic Extract

The NISCAIR authenticated dried *R. serpentina* root sample (voucher specimen number RS1-USBT stored in School of Biotechnology, GGS Indraprastha University) was infused in 1:1 ratio of ethanol and freshly boiled distilled (de-ionized) water. The infusion was kept in shaker at 40 °C for 48 hrs and filtered. The supernatant was freeze dried (lyophilized) in lyophilizer for 24 h. The yield obtained was calculated and freeze-dried aliquots were sealed properly to avoid any moisture inside the vials and stored in deep freezer (−20 °C) till further use.

### 4.3. Liquid Chromatography-Mass Spectrometry

*R. serpentina* hydro-alcoholic root extract was analyzed using various mobile phase combinations for best possible resolution of chromatographic peaks. The LCMS run condition was tested four times for a standardized protocol development to estimate secondary metabolites profile (Table 7).

### 4.4. Reverse Phase-High Pressure Liquid Chromatography

Novel RP-HPLC methods were developed for RES and AJM and validated according to ICH guidelines [13]. HPLC was carried out in Dionex (Thermo scientific, New Delhi, India) system and analyzed by Chromeleon 6.8 chromatography data system software (Thermo scientific, New Delhi, India). The chromatographic conditions used for rapid and precise detection of the compounds are mentioned in Table 8. Parameters of validated method were calculated as linearity, precision, LOD, and LOQ.

RES and AJM present in *R. serpentina* extract were quantified from the pure standards (std) run under same chromatographic conditions.

Quantity (test) = [Average area (test)/Average area (Std)] × [weight·(std)/weight·(test)] ×·Std purity%

where average area is the chromatographic area under the peak for standard and test, weight is measured amount used to prepare dilution.

### 4.5. Preparation of Aβ42 Fibrils

44 µM of Aβ42 was dissolved in milliQ water and mixed with various concentrations of compounds to study. Aβ42 alone sample was used as control. Samples were incubated at 37 °C at 260 rpm for 48 h in the shaker incubator. Samples were taken out at their stipulated time interval and various experimentations (ThT-binding measurement, Congo red, CD spectra analysis, and TEM) were performed to study the inhibition of amyloid fibril formation by the compounds under study.

### 4.6. ThT Fluorescence Spectroscopy Measurements

Aβ42 samples were incubated with or without compounds at different concentrations, after 48 h of fibril formation samples from each experimental set were withdrawn and mixed with ThT in opaque micro titer plate. The reactions were then incubated in dark at 37 °C for 30 min for ThT probe to bind the formed amyloid fibrils. The ThT was excited at 450 nm and spectra were recorded from 400 to 650 nm [27]. All measurements were performed in triplicates and all experiments were repeated thrice individually.

### 4.7. Congo Red Binding Assay

Congo red (200 μM) was dissolved in a 50 mM phosphate buffer (pH 7.4) consisting of 50 mM NaCl and 10% ethanol and filtered through 0.45 µm CA membrane filter. The Aβ42 concentration was fixed at 10 µM in the reaction mixture. CR and protein were mixed at a molar ratio of 1:1 for various experimental setups and kept for 30 min incubation at room temperature. The absorbance spectra (400–650 nm) of the samples were recorded with a UV-visible spectrophotometer [27]. All experiments had three technological and three biological repeats.

### 4.8. Circular Dichroism Spectroscopy 

Secondary structure of Aβ42 peptides were recorded as far UV circular dichroism spectra using Chirascan (Applied PhotoPhysics, New Delhi, India) instrument and software. 22 μM Aβ42 samples (22 μM Aβ42 alone and Aβ42 with RES and AJM) were mixed well ensuring a homogenous suspension and transferred to quartz cell for CD spectra scanning. CD spectra was recorded from 200–260 nm at RT using a bandwidth of 1.0 nm, times per point 0.5 sec, a step interval of 1 nm, and 10 mm path length. Five scans of each sample were measured and averaged. Averaged control buffer scans were subtracted from the sample spectra. The results of CD measurement were plotted as graph between measured ellipticity (millidegree) and wavelength (nm). The secondary structure contents (α-helix, β-sheet, turn, and random coil) of the Aβ42 peptide samples were estimated from the CD spectra using the CD Pro software [20]. Relative percentage of reduced β-sheet content in Aβ42 samples treated with RES and AJM was calculated using BeStSel software [28].

### 4.9. Transmission Electron Microscopy

5 μL Aβ42 prepared fibril samples (22 μM) Aβ42 alone and Aβ42 with RES and AJM) were loaded on carbon stabilized, formvar coated grid for 40 sec. Ashless filter paper was used to wipe out extra sample from the edges of the grid. Loaded grid was negatively stained with 1% uranyl acetate for 20 sec and air-dried. Grid was then examined and photographed on JEOL 2100F transmission electron microscope.

### 4.10. Aβ42 Induced Neurotoxicity in PC12 Cells

Undifferentiated PC12 cell line used as an AD model to study neurotoxicity in the current study, was maintained as monolayer adherent cultures in RPMI 1640 medium supplemented with 5% FBS and 1% antibiotic [29]. Cells were seeded in 96 well plate in log phase of growth at concentration 5 × 10^5^ cells per well. Pre-incubation was given with homotaurine (40 μM, as positive control), RES (10, 20, 40 μM), AJM (10, 20, 40 μM), and *R. serpentina* extract (25, 50, 100 μg/mL). Cytotoxicity with preformed Aβ42fibrils aged 48 h at 40 μM (IC_50_ value) was induced to compounds pre-acclimatized PC12 cells for 24 h. MTT solution was added to the wells and incubated in dark, for the live cells to convert the tetrazolium salt in purple formazan crystals. The formazan crystals were dissolved using DMSO and absorbance was measured at 570 nm. Relative survival percentage of PC12 cells was calculated compared to control (PC12 cells only). All measurements were performed in triplicates and all experiments were repeated thrice individually.

### 4.11. Neuroprotection Against Oxidative Stress Cytotoxicity Using H_2_O_2_

Oxidative stress was induced to cultured PC12 cells using hydrogen peroxide (IC_50_ 200 μL) and concentrations of glutathione (positive control), RES, AJM (10, 20, 40 μM), and *R. serpentina* extract (25, 50, 100 μg/mL) were used to analyze neuroprotective effects against oxidative damage through MTT assay. Cell viability was determined through trypan blue dye exclusion assay and cells were seeded to 96 well tissue culture plate at a 5 × 10^5^ cells/well concentration. Pre-incubation with compounds were given to PC12 cells prior to inducing H_2_O_2_ cytotoxicity for 18 h. MTT was added to the well at 5 mg/mL concentration and incubated in the dark for enzymatic reaction by live cells for 2 h. DMSO was added to well to dissolve formazan crystals and absorbance was recorded at 570 nm. PC12 cells relative survival was calculated compared to control (PC12 cells only).

### 4.12. In Vitro ChE Enzyme Assay

Ellman assay was performed to assess the inhibition of the enzyme AChE (EC 3.1.1.7) and BuChE (EC 3.1.1.8) by donepezil (positive control), RES, AJM, and *R. serpentina* extract [30]. Five microliters of enzyme (AChE and BuChE) solution at 0.03 U/mL final assay concentration; 200 μL of phosphate buffer (pH 8); 5 μL of DTNB (0.3 mM); and 5 μL of test compounds were pre-incubated for 15 min at RT. The reaction was initiated by adding 5 μL of substrate (ATChI and BuTChI) at a final concentration of 0.5 mM. The control for relative absorbance consisted of the buffer in place of the test compounds and the enzyme and substrate blank were devoid of enzyme and the substrate, respectively. Three biological and three experimental repeats were performed and read at 412 nm on a SpectraMax M2 reader.

### 4.13. In Vitro BACE-1 Enzyme Inhibition Assay

BACE-1 (EC 3.4.23.46) activity inhibition was assayed using FRET (fluorescence resonance energy transfer) kit (Sigma Aldrich, New Delhi, India). With all reaction components at RT, 50 μM substrate was added to 75 μL assay buffer containing RES and AJM (25, 50, 100 μM) in the microtiter plate. Reaction was initiated by the addition of 0.03 U BACE-1 enzyme in the well and zero minute fluorescence was recorded (Ex 350 nm, Em 405 nm). After 2 h incubation in dark at 37 °C, reaction fluorescence was recorded every one hour for next three hours. Relative fluorescence inhibition was calculated from zero minute fluorescence reading as control. Three individual experiments were performed.

### 4.14. Monoaminoxidase-B (MAO-B) Inhibition Assay

MAO-B (EC 1.4.3.4) activity inhibition was assayed using Biovision inhibition screening kit (fluorometric). With all reaction components at room temperature, 0.2 U enzyme was pre-incubated with compounds under study and incubated for 10 min. Working substrate was prepared fresh by adding developer and oxired probe with assay buffer according to kit instructions. Substrate was added to the reaction and incubated for 10 min; zero-minute fluorescence was recorded (Ex 535 nm, Em 587 nm) to serve as control for relative fluorescence inhibition calculation. All measurements were performed in triplicates and all experiments were repeated thrice individually.

### 4.15. Molecular Docking Analysis by Autodock 4.2

Molecular interactions of RES and AJM with selected AD targets (Aβ42, AChE, BuChE, BACE-1, and MAO-B) were studied using Autodock 4.2 [31]. Respective of good resolution, co-crystallized structures were procured from protein data bank (1YIT for Aβ42, 4PQE for AChE, 2J4C for BuChE, 4D8C for BACE-1, and 1S2Q for MAO-B). Three-dimensional structures of RES and AJM and known inhibitors (galanthamine, tacrine, rasagilline, tannic acid, and BXD) were downloaded from Pubchem Database. Co-crystallized proteins were processed using PyMol to get the Apo structure of the protein and ligand in separate formats [32]. Re-docking was done with prepared co-crystalized ligand and prepared receptor protein to validate the docking protocol and the maps were generated. Root mean square deviation (RMSD) was calculated as a measure of legitimate docking protocol. Post validation of the docking protocol, RES and AJM processed ligand structures were individually docked with target receptor proteins in the study. Molecular interactions, ligand conformations, and binding energies were obtained.

### 4.16. Pharmacokinetic Analysis (ADMET)

ADMET analysis was carried out using Drulito software (www.niper.gov.in/pi_dev_tools/DruLiToWeb/DruLiTo_index.html) to study ideal pharmacokinetics profile of RES and AJM for drug development. Two filters were used for screening; Lipinski rule and blood brain barrier. Lipinski rule states that for an ideal drug molecule should weigh below 500 g/mol, hydrogen bond donor should be less than or equal to 5 and number of hydrogen bond acceptor should be less than or equal to 10 along with a partition coefficient of 5 or less. Such compounds would pass the BBB if the number of hydrogen bonds present is below eight and there should not be any acidic group present in the molecule. Total polar surface area (TPSA) showed bioavailability of the drug molecule as per Veber’s rule the TPSA less than or equal to 140Å indicates good oral bioavailability.

### 4.17. Statistical Analysis

Three independent experiments and each experiment with three replicates, were used in each experimental setup to present data as mean ± standard error mean but for LCMS data *n* = 4 runs were used. Statistical analysis was performed using one-way ANOVA for comparing independent means, followed by Bonferroni post test for multiple comparisons of experimental setups with control groups. Level of significance was determined as *p* ≤ 0.05 and *p* < 0.01, until otherwise stated.

## 5. Conclusions

In conclusion, the novel study showed that RES and AJM could act as multi-target directed ligands and can be used to develop into novel compound that can be used against several targets Aβ42, AChE, BuChE, MAO-B, BACE-1, and ROS that are implicated in AD and thus, help in alleviation of symptoms and having disease modifying effect in AD.

## Figures and Tables

**Table 1:** List of identified compounds in electron spray ionization (ESI) positive mode of ionization from *R. serepentina* hydroalcoholic extract by UHPLC-QTOF analysis.

Peak No.	Compound Name	Formula	Monoisotopic Mass	Retention Time (min)	*m* + *z* Values
1	Behenic Acid	C_22_H_44_O_2_	340.592	5.7	341.578
2	Sarpagine	C_19_H_22_N_2_O_2_	310.168	8.5	311.168
3	Ajmaline	C_20_H_26_N_2_O_2_	326.199	12.9	327.199
4	Ajmalicine	C_21_H_24_N_2_O_3_	352.179	13.4	353.177
5	Yohimbine	C_21_H_26_N_2_O_3_	354.194	13.7	355.196
6	Serpentine	C_21_H_20_N_2_O_3_	348.147	14.6	349.148
7	Reserpine	C_33_H_40_N_2_O_9_	608.273	15.3	609.271
8	Deserpedine	C_32_H_38_N_2_O_8_	578.263	15.9	579.263
9	Reserpiline	C_23_H_28_N_2_O_5_	412.2	16.3	413.202
10	Alpha or Beta Amyrin	C_30_H_50_O	426.291	18.6	427.292
11	Indobine	C_11_H_11_NO_2_	119.073	19.2	120.083
12	Rutin	C_27_H_30_O_16_	611.52	20.6	612.521
8	Unknown_RS1			15.9	290.264
9	Unknown_RS2			16.3	653.239
11	Unknown_RS3			19.2	381.288
13	Unknown_RS4			20.6	685.422

**Table 2:** Characteristics of reverse phase HPLC (RP-HPLC) method development and validation for reserpine (RES) and ajmalicine (AJM).

Indole Alkaloids	t_R_ * (min)	Peak Asymmetry	RSD (%)	S/N LOD (ppm)	S/N LOQ (ppm)	Linear Regression Equation (Y = AX + C)
Ajmalicine	6.1 ± 0.2	0.91	2.3	3.7	10.5	y = 1.5808 x + 0.0495 R² = 0.99984
Reserpine	5.3 ± 0.1	0.94	1.8	2.82	9.76	y = 130306 x − 5265.8 R² = 0.99998

Peak asymmetry should be around one; S/N—signal to noise ratio; ppm—parts per million; RSD—relative standard deviation; and *n* = 6 repeated analysis, * t_R_—retention time represented as mean ± SD.

**Table 3:** Estimated secondary structure content (%) using BeStSel (Beta structure selection) software from CD spectra.

Secondary Structure	Aβ42 Control (%)	Aβ42 + RES (%)	Aβ42 + AJM (%)
α-helix	0.0	0.0	0.0
β-sheet (antiparallel)	74.9	27.3	35.2
β-sheet (parallel)	16.5	0.0	0.0
Turn	8.6	17.8	11.7
Others	0.0	54.9	53.1

**Table 4:** Inhibitory activity (IC_50_) and selectivity index (SI) of the compounds investigated against AChE and BuChE.

Compound	IC_50_ (μM ± SD)/IC_50_ (µg/mL ± SD)		SI
	AChE IC_50_	BuChE IC_50_	
Reserpine	1.7 ± 2.08 µM	2.8 ± 1.84 µM	1.65
Ajmalicine	3.5 ± 1.41 µM	5.44 ± 1.75 µM	1.55
*R. serpentina* extract	14 ± 3.62 µg/ml	22 ± 3.10 µg/ml	1.57
Donepzil	0.98 ± 1.20 µM	1 ± 2.89 µM	1.02

Regression analyses was used to determine IC_50_ values were and expressed as the means ± SD of three replicate determinations. Selectivity index here is the AChE selectivity over BuChE defined as IC_50_ BuChE/IC_50_ AChE affinity ratio.

**Table 5:** Molecular interactions as observed in ligand docked complexes of target receptor proteins obtained from Autodock 4.2.

S.No.	Ligand	AD Target	Binding Energy (Kcal/mol)	No. of H-bonds	Interacting Residues	Bond Angle (Å)
1.	RES (Reserpine)	Aβ42	−9.45	5	Asp23, Gly33, Lys28, Leu34, Val36	3.2, 2.4, 2.4, 3.3, 3.3, hydrophobic with Lys28
		AChE	−11.42	7	Phe295, Arg296, Tyr337, Ser125, Glu334, Tyr72	2.3, 3.5, 1.9, (2.6, 2.3), 2.4, 2.7
		BuChE	−7.68	4	Asn68, Thr120, Ala277, Val288	3.4, 2.3, 3.4, 2.4
		BACE-1	−8.8	5	Thr72, Asp32, Asp217,	2.6, (3.2, 3.3), (3.5, 3.0)
		MAO-B	−3.7	3	Val85, Tyr326, Ile199	3.4, 2.4, 2.8
2.	AJM (Ajmalicine)	Aβ42	−8.4	1	Asp 23	3.2
		AChE	−9.7	3	Phe295, Tyr286, Phe297	2.1, 3.0, 3.2
		BuChE	−6.6	4	Asn68, Asp70, Trp82, Thr120	3.6, 2.1, 2.5, 2.1
		BACE-1	−8.9	0 (all hydro-phobic)	Asp32, Asp228	
		MAO-B	−5.6	6	Glu85, Pro102, Thr202, Glu84	3.3, 2.4, 2.1, (3.2, 3.3)
Positive Control						
3.	Tannic acid	Aβ42	−6.5	2	Asp23, Lys28	2.1, 1.9
4.	Galanthamine	AChE	−10.8	3	Tyr337, Glu202, Ser203	1.8, 2.2, 1.7
5.	Tacrine	BuChE	−6.52	1	Trp82	2.3
6.	BXD	BACE-1	−10.4	4	ASP32, GLY34, PHE108, ASP217	1.9, 2.2, 2.5, 2.1
7.	Rasagilline	MAO-B	−7.5	0 (all hydro-phobic)	Gln206, Phe343, Tyr326, Leu171	

**Table 6:** Pharmacokinetics ADMET prediction by Drulito against Lipinski rule of five and blood-brain-barrier filter.

Compound	MW (g/mol)	logP	AlogP	HBA	HBD	TPSA	nHB	nAcidic group	Filter L/B
Reserpine	608.27	2.672	−0.857	11	1	114.02	12	0	B
Ajmalicine	352.18	1.906	−0.068	5	1	50.8	6	0	L/B
Tacrine	198.12	1.121	−0.748	2	1	38.38	3	0	L/B
Galanthamine	287.15	1.197	−0.444	4	1	41.93	5	0	L/B
Donepezil	379.21	2.633	0.364	4	0	38.77	4	0	L/B
Rasagiline	171.1	1.446	1.092	1	1	12.03	2	0	L/B
Tannic acid	1701.17	9.537	−5.356	46	25	777.9	71	0	

MW—molecular weight; logP—partition coefficient; AlogP—octanol–water partition coefficient; HBA= hydrogen bond acceptor; HBD—hydrogen bond donor; TPSA—total polar surface area; nHB—number of hydrogen bond; nAcidic group—number of acidic group; Filter L—Lipinski rule of five; and B—blood brain barrier.

**Table 7:** Optimized UHPLC-QTOF condition for identification of phytoconstituents present in *R. serpentina* extract.

Variables	Conditions
System	Agilent 6520 QTOF LC/MS system
Software	Agilent MassHunter B.05.00
Column	Zorbax UPLC C_18_ (100 mm × 2.1 mm, i.d. 1.7 µm)
Mobile phase	Water with 0.01% formic acid (A) and methanol (B)
Flow Rate	0.4 mL/min
Fragmentor voltage	150 V
Electron spray ionization (ESI) mode	Positive
Mass-to-charge (*m*/*z*) scanning ratio	100–1000 *m*/*z*
Injection volume	3 µL
Column Temperature	25 °C
Elution	Linear gradient: 0–5 min, 10–20% B 5–14 min, 20–30% B 14–18 min, 30–75% B 18–22 min, 75–100% B 22–31 min, 10 %B

Data analysis: The LCMS data was analyzed by Mass Hunter software developed by Agilent. Peaks generated in positive mode of ionization above ≥2000 counts were considered with peak spacing tolerance of 0.0025 *m*/*z* for better resolution of LC/MS. Mass Bank Workstation software along with METLIN database was used to estimate metabolite profile [26].

**Table 8:** Chromatographic conditions for RES and AJM RP-HPLC method with UV detection.

Variables	Conditions for RES	Conditions for AJM
Absorbance maxima (λ)	260 nm	268 nm
Column	Zorbax HPLC C_18_ (150 mm × 4.6 mm, i.d. 5 µm)	Inertsil HPLC C_8_ (250 mm × 4.6 mm, i.d. 5µm)
Mobile phase	Water:Methanol (20:80 *v*/*v*)	Water:Acetonitrile (30:70 *v*/*v*)
Flow Rate	1 mL/min	0.5 mL/min
Injection volume	10 µl	20 µl
Elution	Isocratic	Isocratic

## Abbreviations

AD—Alzheimer’s disease, RES—Reserpine, AJM—Ajmalicine, MTDL—Multi-target directed ligand, APP—Amyloid precursor protein, Aβ42—Amyloid beta 1-42 residues, AChE—Acetylcholinesterase, BuChE—Butyrylcholinesterase, MAO-B—Monoaminooxidase B, BACE1—Beta site cleaving enzyme 1(beta—secretase), ROS—Reactive oxygen species, CAS—Catalytic anionic site, PAS—Peripheral anionic site, LOD—Level of detection, LOQ—Level of quantification, UPLC—Ultra pressure liquid chromatography, QTOF—Quadrupole time of flight, HPLC—High pressure liquid chromatography, ThT—Thioflavin T, CR—Congo Red, CD—Circular dichroism.
